# Transplantation after Mustard operation for transposition of the great arteries

**DOI:** 10.1002/ccr3.4930

**Published:** 2021-10-10

**Authors:** Shintaro Katahira, Yukiharu Sugimura, Hug Aubin, Hayato Ise, Yoshikatsu Saiki, Artur Lichtenberg, Ralf Westenfeld, Udo Boeken, Payam Akhyari

**Affiliations:** ^1^ Department of Cardiac Surgery Medical Faculty Heinrich‐Heine‐University Düsseldorf Germany; ^2^ Department of Cardiothoracic Surgery University Hospital Tohoku University Sendai Japan; ^3^ Department of Cardiology, Angiology and Pulmology Medical Faculty Heinrich‐Heine‐University Düsseldorf Germany

**Keywords:** d‐TGA, heart transplantation, heart failure, mustard

## Abstract

As long‐term outcomes of congenital heart diseases improve, the probability of adult patients presenting for heart transplantation for late failure of congenitally corrected heart disease also increases. In patients with dextro‐transposition of the great arteries (d‐TGA) who were initially treated in the era of Mustard or Senning procedures and before Jatene procedure was introduced, progressive systemic right ventricular failure represents a problem in the very long‐term follow‐up. We report a rare case of heart transplantation as a third operation 36 years after Mustard procedure in a patient with d‐TGA experiencing late failure of the systemic right ventricle.

## Graphical abstract

Heart transplantations are becoming more common after surgery for congenital heart disease. Many heart transplantations have been performed also after Mustard surgery, but few have reported detailed surgical procedures with figures or video. This report is useful because of the variations in d‐TGA.

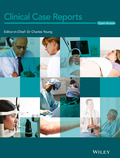

## 1 INTRODUCTION

In dextro‐transposition of the great arteries (d‐TGA), the Jatene procedure was introduced in 1975, resulting in greatly improved outcomes ^1^. However, failure of the systemic right ventricle (sRV) in adulthood becomes a problem in earlier types of correction, for example, Mustard or Senning procedure ^2^. In the latter patient cohort, heart transplantation (HTX) is the only treatment for sRV failure. Intraoperative identification and orientation regarding the atrial chambers, as well as anatomic variation with respect to the position of the great arteries, represent technical challenges during HTX. Current literature contains few narrative reports on HTX after Mustard procedure ^3^, ^4^. Here, we demonstrate a case along with a surgical video of successful HTX after Mustard operation.

## 2 CASE REPORT

**FIGURE 1:**
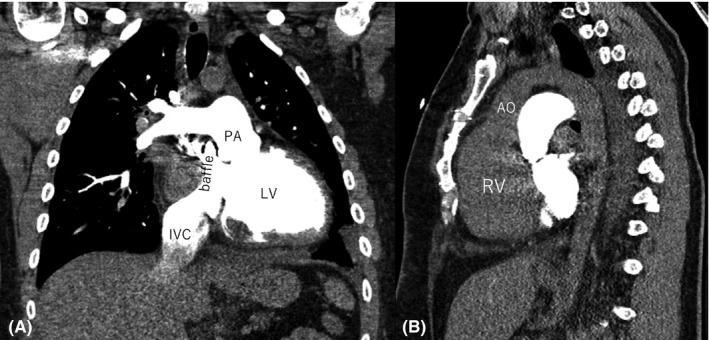
Preoperative enhanced computed tomography. A, Frontal view demonstrates that blood flow of SVC and IVC are redirected through the baffle toward the mitral valve and the LV connected with PA. B, Sagittal view shows that the systemic RV and aorta are located anteriorly with respect to the pulmonary ventricle and the pulmonary trunk. PA, pulmonary artery; LV, left ventricle; IVC, inferior vena cava; SVC, superior vena cava; RV, right ventricle

A 36‐year‐old man with d‐TGA and Mustard procedure at 15 months after birth and second operation with augmentation plasty for SVC stenosis at the age of 6 years was admitted to the emergency department for heart failure (HF) symptoms. On echocardiography, sRV was dilated (end‐diastolic dimension 56mm) with a severely impaired ejection fraction (EF) of 15% and moderate regurgitation at systemic atrioventricular valve. Preoperative computed tomography showed anterior location of the aorta, regular anatomic relation of the Mustard baffle in the systemic atrium in close anatomic relation to the pulmonary valve, moreover the anterior wall of the systemic ventricle grossly adhering to the dorsal aspect of the sternum (Figure 1A, B). Pulmonary artery catheterization showed low cardiac index (1.65 l/min*m^2^) and pulmonary hypertension (mean 40 mmHg). Initial lactate was 1.6 mmol/l. Furthermore, NT‐proBNP was significantly elevated to 6792 pg/ml, as also serum creatine (1.4 mg/dl), and total bilirubin (1.4 mg/dl). The patient was admitted to intensive care and high urgency (HU) status was granted. Fifty‐two days after HU status, HTX was performed 35 years after Mustard procedure.

**FIGURE 2:**
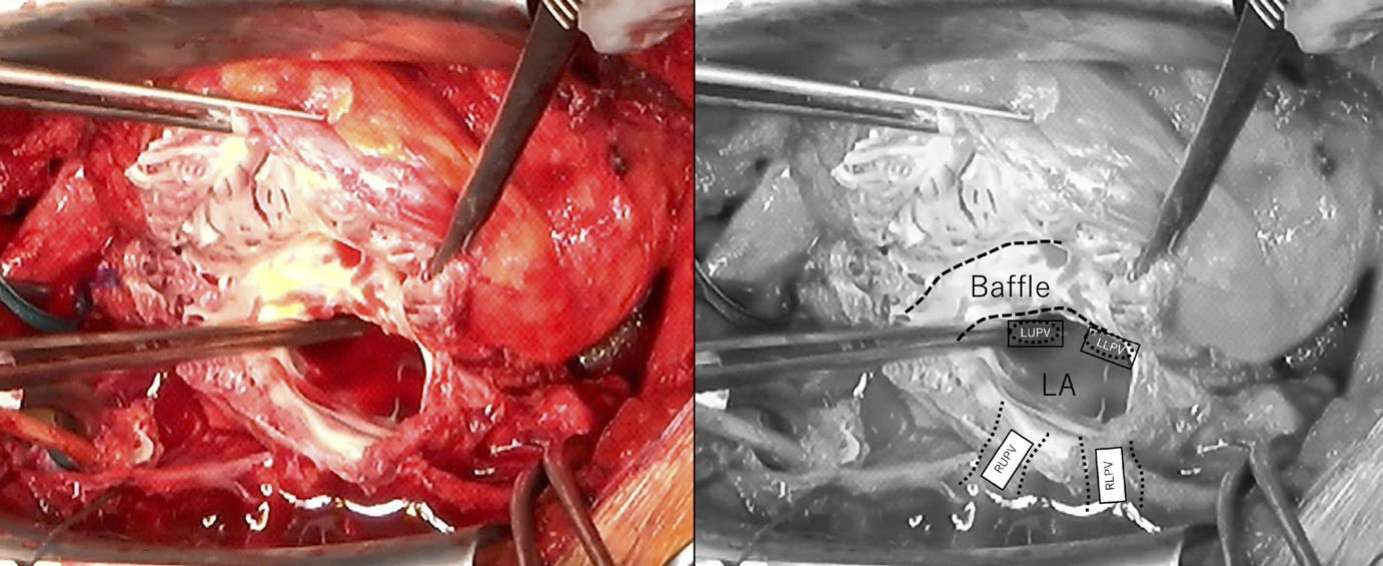
Intraoperative imaging. When the anatomic right atrium, that is, systemic atrium, was incised, the Mustard baffle became immediately identified and LA and PV could be confirmed underneath in the depth of the systemic atrial chamber. LA, left atrium; LUPV, left upper pulmonary vein; LLPV, left lower pulmonary vein; RUPV, right upper pulmonary vein; RLPV, right lower pulmonary vein

The operative strategy included femoral cannulation and cardiopulmonary bypass (CPB) initiation prior to resternotomy due to expected adhesions to sRV. After partial dissection of the adhesions, the systemic atrium was vented to avoid pulmonary congestion during further preparation and manipulation. Aortic clamping was performed early to avoid air embolism, and both caval veins were incised for later bicaval implantation of donor heart. Next, systemic (ie, anatomic right) atrium was opened and Mustard baffle and pulmonary vein (PV) ostia were identified from the endocardial side as well as PV location from outside of the atrium (Figure 2). Aorta and pulmonary trunk were transected more distally than in regular HTX to achieve a more regular anatomic relation. The resulting recipient dimension of the aorta was remarkably small. Due to the specific technique of Mustard correction, the inter‐PV distance revealed to be relatively small and the anatomic left atrial cuff limited in size when compared to the common anatomy in HF patients. Therefore, when excising recipient heart, the incision line was performed as much as possible distant from the PV ostia. Additional perpendicular incision of the remaining left atrial cuff was performed in between the two left PV ostia as well as caudally and cranially in between the left and right atrial PV ostia to enlarge the anastomotic line on the recipient side. As a further modification on the donor side, cardiac graft was harvested with a long segment of the aorta, including most part of the aortic arch. HTX was performed by bicaval method, and the anastomosis was performed in the order of left atrium, IVC, SVC, pulmonary artery, and ascending aorta. Despite the more liberal excision of recipient great arteries, the distal ascending aorta was yet located anteriorly and slightly left to the normal anatomy. Utilizing longer segments of the donor graft and more distal anastomotic lines, it was possible to perform both anastomoses of great arteries without the use of prosthetic materials. The aortic anastomosis was further complicated by a remarkable size mismatch but proved to be feasible without prosthetic material. Total donor heart ischemia time was 214 minutes. After 131 min of reperfusion, weaning from CPB was performed with moderate doses of catecholamines, inhalative nitric oxide, and intermittent inhalative prostacyclin therapy. The patient was extubated on the 1^st^ postoperative day, and further postoperative course was unremarkable. There was no particular problem with postoperative echocardiography, and he was discharged on the 33^rd^ postoperative day without any other complications.

## 3 DISCUSSION

Mustard procedure was the preferred type of surgery that was performed on d‐TGA before the Jatene procedure was introduced and widely adopted.^2^ The problem of Mustard procedure is that by preserving the anatomic right ventricle for systemic circulation late postoperative dysfunction and failure of the sRV is a common complication. Implantable ventricular assist device (VAD) therapy has been reported in the latter scenario, however, with mixed results of VAD in sRV, which may suggest to favor HTX as the first choice in this particular patient cohort ^5^.

Beyond pre‐operative stabilization and postoperative management, intraoperative technical issues represent important components for successful treatment of d‐TGA patients with failing systemic ventricle. Anatomic abnormalities represent a considerable challenge, particularly after previous operations. A thorough diagnostic workup utilizing modern imaging modalities for precise localization of native structures (eg, course and ostia of PVs) and reconstructive implant material (eg, atrial baffle) should be obtained to improve the quality of preoperative decision finding regarding operative strategy. Although not experienced in this case, further reconstruction steps may be necessary for complex anatomic scenarios to enable HTX, for example, using a baffle or vascular grafts ^3^.

The point devised in the surgery of this case was the creation of the left atrial anastomosis. Since the adhesion between the right atrium and PV was severe, ascending aorta was clamped, the right atrium was incised to confirm the left atrium and PV from the atrium and pericardium, and the heart can be removed safely without injury. In this case, IVC and SVC were located on the right side, no special reconstruction was required, and reconstruction with the normal bicaval method was possible.

## CONFLICTS OF INTEREST

The authors declare no conflicts of interest associated with this manuscript.

## AUTHOR CONTRIBUTIONS

Shintaro Katahira and Payam Akhyari: designed research/study. Shintaro Katahira: drafted the manuscript. Yukiharu Sugimura, Hug Aubin, Hayato Ise, Yoshikatsu Saiki, Artur Lichtenberg, Ralf Westenfeld, Udo Boeken, and Payam Akhyari: critically revised the manuscript. Artur Lichtenberg, Udo Boeken, and Payam Akhyari: involved in responsibility for treatment decisions. Yoshikatsu Saiki, Artur Lichtenberg, Udo Boeken, and Payam Akhyari: supervised the manuscript.

## ETHICAL APPROVAL

This manuscript followed the principles of the Declaration of Helsinki and the Declaration of Istanbul.

## CONSENT

The appropriate informed consent was obtained for the publication of this manuscript.

## Supporting information

Video S1

## Data Availability

Data sharing is not applicable to this article as no new data were created or analyzed in this study.
